# Temporal Gene Expression Variation Associated with Eyespot Size Plasticity in *Bicyclus anynana*

**DOI:** 10.1371/journal.pone.0065830

**Published:** 2013-06-10

**Authors:** Jeffrey C. Oliver, Diane Ramos, Kathleen L. Prudic, Antónia Monteiro

**Affiliations:** 1 Department of Ecology and Evolutionary Biology, Yale University, New Haven, Connecticut, United States of America; 2 Zoology Department, Oregon State University, Corvallis, Oregon, United States of America; 3 Natural Sciences Department, Daemen College, Amherst, New York, United States of America; University of Otago, New Zealand

## Abstract

Seasonal polyphenism demonstrates an organism's ability to respond to predictable environmental variation with alternative phenotypes, each presumably better suited to its respective environment. However, the molecular mechanisms linking environmental variation to alternative phenotypes via shifts in development remain relatively unknown. Here we investigate temporal gene expression variation in the seasonally polyphenic butterfly *Bicyclus anynana*. This species shows drastic changes in eyespot size depending on the temperature experienced during larval development. The wet season form (larvae reared over 24°C) has large ventral wing eyespots while the dry season form (larvae reared under 19°C) has much smaller eyespots. We compared the expression of three proteins, Notch, Engrailed, and Distal-less, in the future eyespot centers of the two forms to determine if eyespot size variation is associated with heterochronic shifts in the onset of their expression. For two of these proteins, Notch and Engrailed, expression in eyespot centers occurred *earlier* in dry season than in wet season larvae, while Distal-less showed no temporal difference between the two forms. These results suggest that differences between dry and wet season adult wings could be due to a delay in the onset of expression of these eyespot-associated genes. Early in eyespot development, Notch and Engrailed may be functioning as repressors rather than activators of the eyespot gene network. Alternatively, temporal variation in the onset of early expressed genes between forms may have no functional consequences to eyespot size regulation and may indicate the presence of an 'hourglass' model of development in butterfly eyespots.

## Introduction

Phenotypic plasticity occurs when identical genotypes develop different phenotypes upon exposure to different environmental conditions. Examples include the different mating behaviors in crickets [1]; caste determination in ants [2], and horn length in dung beetles [3]. Seasonal polyphenism, a form of phenotypic plasticity, is the phenomenon where predictable environmental variation leads to the development of distinct, presumably adaptive, phenotypes in the respective environmental conditions. The molecular mechanisms whereby this environmental variation is translated into phenotypic differences remains a key question in developmental biology [4].

*Bicyclus anynana* (Butler) (Lepidoptera: Nymphalidae) is a model system for the study of seasonal polyphenism and demonstrates striking phenotypic plasticity in wing morphology in the cohorts that emerge during the dry season (DS) and the wet season (WS) [5], [6]. When larvae are reared in warm conditions (corresponding to wet season temperatures), adult *B. anynana* have large, conspicuous eyespots on the ventral border of the fore- and hindwings. In contrast, larvae reared under cool conditions (corresponding to dry season temperatures) become adults with highly reduced ventral wing eyespots. The sensitive period for this change in phenotype appears to be in the fifth (ultimate) instar of larval development [7]. Ecdysteroid signaling may mediate the plasticity of eyespot size [8], [9], but little is known about how this signal is translated into variation in gene expression for those genes expressed in developing eyespots.

Several genes are expressed in the future eyespot centers of fifth instar *B. anynana* wing discs [10], , including those coding for the transcription factors Engrailed (En) [12] and Distal-less (Dll) [13], and the trans-membrane receptor protein Notch (N) [14]. These genes are expressed in a group of signaling cells, the focus, responsible for differentiating the eyespot via the action of one or more hypothesized morphogens [15], [16], [17], [18].

Changes in expression dynamics caused by rearing temperature present a potential mechanism by which these three genes may affect eyespot size plasticity. Expression levels of all three genes are positively correlated to eyespot size when butterflies are reared at a constant temperature [14], [19], [20], suggesting that quantitative differences in gene expression could be mediating the temperature-driven size change in the seasonal forms. Additionally, the expression level of Dll during pupal development is positively correlated with eyespot size in response to rearing temperature in WS and DS forms [13], and transgenic manipulation of Dll expression in fifth instar larvae results in changes in adult eyespot size [21]. A heterochronic shift in the establishment of the signaling focus could lead to differences in expression levels of these genes between the two seasonal forms. Heterochronic shifts in gene expression can lead to differences in trait size (e.g., *Sonic hedgehog* expression in fin development, [22]. Furthermore, temperature experienced during development can lead to heterochrony, ultimately resulting in alternative phenotypes [23]. Heterochrony in gene expression may underly the differences between the two seasonal forms of *B. anynana*; however, this has not yet been investigated in regards to plasticity in eyespot size.

Here we investigate the link between environmental variation and temporal differences in gene expression in the eyespot foci of the two seasonal forms of *B. anynana*. We test whether rearing temperature during larval development alters the onset of gene expression in the eyespot foci of DS and WS forms. Such a shift in the timing of focal expression could either delay or accelerate focal differentiation, thereby impacting focal signaling, and ultimately leading to changes in eyespot size. We used controlled laboratory rearing conditions to test the hypothesis that smaller eyespots in the DS are associated with delayed expression of the proteins N, En, and Dll.

## Materials and Methods

### Specimens and Immunohistochemistry

*Bicyclus anynana* larvae were reared from the Yale colony established from Malawi. Larvae were reared under one of two conditions, differing only in rearing temperature. The WS-inducing temperature was 27°C and the DS-inducing temperature was 17°C; all larvae were reared at 80% RH, 12hr light:dark cycle. We collected fifth instar larvae and fixed wing discs following the protocol of [24]. Discs were stained for En (4F11 mouse monoclonal anti-en at 1∶5; N. Patel), Dll (rabbit polyclonal anti-Dll at 1∶200; G. Boekhoff-Falk), or N (C17.9C6-s mouse monoclonal anti-Notch at 1∶20). We used donkey anti-mouse (Jackson Immunoresearch #715-095-150) and goat anti-rabbit (Molecular Probes #T-2767) secondary antibodies at a concentration of 1∶200. All wings were mounted with ProLong Gold (Invitrogen, Carlsbad, CA, USA); expression images were captured on a Nikon 90i microscope using the NIS-Elements software (Nikon Instruments, Melville, NY, USA).

### Testing for Temporal Expression Differences

Comparing the timing of gene expression between the two phenotypes requires an internal measure of developmental stage. Because the length of fifth instar development is considerably different between the two rearing conditions (length of fifth instar: WS = 6±1.5 days; DS = 18±2.9 days), absolute time is not appropriate for comparing temporal dynamics of gene expression. We instead used the protocol of [14] to categorize wing developmental stage based on the extent of tracheal growth in the developing wing tissue. Wing discs were scored for their developmental stage and gene expression for each wing compartment that bears an eyespot on the adult wing. This led to a maximum of nine possible compartments scored per individual: the M_1_ and Cu_1_ compartments of the forewing and the Rs, M_1_, M_2_, M_3_, Cu_1_, Cu_2_, and Pc compartments of the hindwing. Each wing compartment was scored as either exhibiting focal expression in the future center of the eyespot (1) or absence of focal expression (0) as described in [25]. We then combined data across wing compartments and analyzed data without regard to compartment identity (i.e. data for all nine wing compartments were combined in a single matrix of developmental wing stage and focal expression for all subsequent analyses).

To test for differences in timing of gene expression between the two forms, we compared logistic models of expression based on observed data to expected logistic models [25]. Briefly, for each gene and each form, we fit a logistic model where developmental stage predicted the dependent binary variable of expression (focus or no focus). We then compared the curves for each gene between forms by calculating the difference in area under each form's curve. This difference was then compared against an expected distribution of differences, based on bootstrapping of the observed data. When the observed difference between the curves fell outside of the 95% distribution, based on 10,000 bootstrap replicates, we inferred significant differences in expression timing between the two forms. A detailed description of the approach can be found in [25].

## Results and Discussion

### Expression Variation

**Figure 1:**
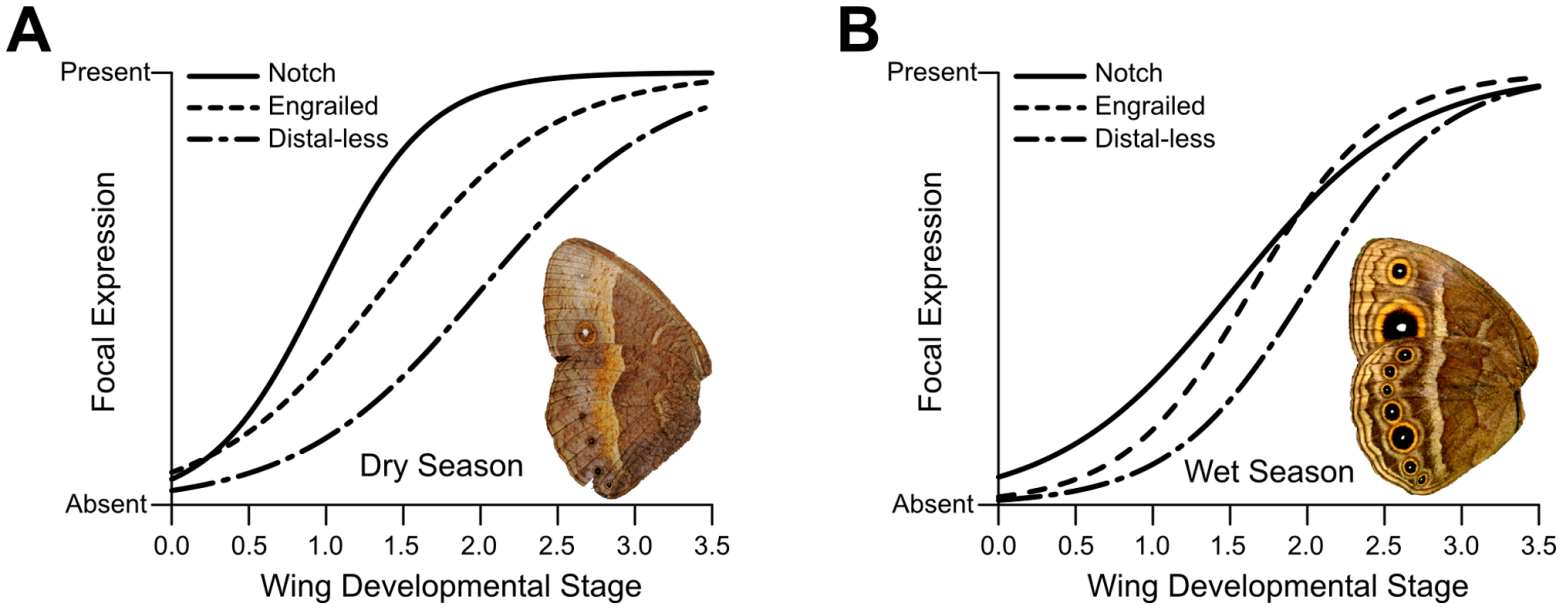
Temporal dynamics of three eyespot-associated genes in different forms of *B. anynana*. In both dry season (A) and wet season (B) larvae, genes are expressed in future eyespot centers in the same order as described in [25].

**Figure 2:**
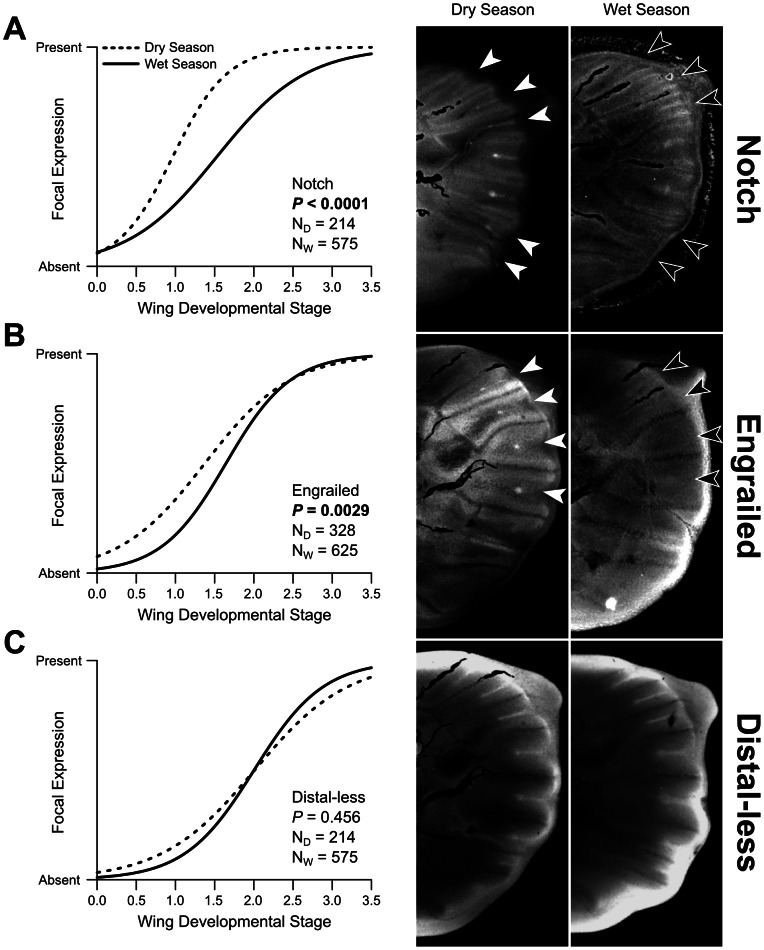
Temporal variation in expression of protein products of three eyespot-associated genes. Comparisons of Notch (A), Engrailed (B), and Distal-less (C) expression in two forms of *B. anynana*. Graphs show logistic curves fit to observed expression in each of the two forms. Images show expression in dry season and wet season wing discs of the same developmental stage; N_D_ and N_W_ reflect total compartments examined in dry and wet season wing discs, respectively. In (A) and (B), white arrows in dry season images indicate compartments with focal expression of respective proteins; black arrows indicate corresponding compartments in wet season wing discs, which lack focal expression at this stage.

We observed the same relative temporal order of focal expression of the three genes in WS and DS wing discs during the sensitive period of larval development (Figures 1 and S1). As shown previously for WS specimens in *B. anynana* and a variety of other nymphalid species [25], focal expression occurs first in N, then En, and finally Dll. Contrary to our prediction, however, expression of N and En in future eyespot centers occurred earlier in larvae raised in DS conditions than in WS conditions (N: P<0.0001; En: P = 0.0029) (Figures 2A and 2B). We detected no temporal differences in Dll expression between the two seasons during the fifth instar (P = 0.456) (Figure 2C), as suggested by previous work [13]. It is important to note that expression domain size differences in Dll between the two seasonal forms have previously been described [13], but only in the pupal stage, well after the developmental sensitive period for eyespot focal establishment and size polyphenism ([13] and [7], respectively). We found early expression of En and N is associated with the development of smaller eyespots. Thus, a delayed onset of eyespot-associated gene expression during the sensitive developmental period is not responsible for the reduced eyespots observed in DS adults.

### Potential Causes of Variation

While we assumed that a late onset of gene expression in eyespot centers would lead to smaller trait size, the opposite pattern was observed. This finding, where early expression leads to a reduction in trait size, is not without precedent. The polyphenic arctic char, *Salvelinus alpinus* (Salmoniformes: Salmonidae), has two benthivorous morphs that differ primarily in size. The smaller, or 'dwarf', morph is characterized by earlier expression of *Pax7* [26]. This gene is hypothesized to regulate segmentation during development [27], and *S. alpinus* segmentation begins earlier in small morphs than in large morphs, although both forms complete segmentation at the same time. In the case of *B. anynana*, the early onset of N and En expression in DS eyespots (Figures 2A and 2B) may indicate that these genes are repressors of the eyespot gene regulatory network, and by being expressed earlier in the DS form lead to a more extensive repression of the network. This early repression may result in fewer cells differentiating as foci and, ultimately, leading to smaller eyespots in adult wings. We were unable to investigate the potential for differences in total area of expression, but future work should investigate the possibility that temporal differences in focal establishment lead to subsequent quantitative differences in total expression area.

Alternatively, the observed differences in the early-expressed proteins N and En, compared with identical expression in Dll, which is expressed later, suggests that eyespots may follow an 'hourglass' model of development [28], and that the early gene expression differences are essentially neutral regarding function. The hourglass model was originally used to describe the observation that development in mid-embryogenesis is conserved across animal phyla, while early and late development are characterized by considerable variation. Variation in gene expression among *Drosophila* species follows this model, where mid-embryogenesis is characterized by the lowest inter-specific temporal variance in gene expression [29]. In this hourglass model, variation mid-development has significant, often deleterious, influence on the final phenotype [28]. The observed variation in eyespot-associated gene expression may not occur at this hypothetical 'waist' stage, and thus does not affect the phenotype of the eyespot. Previous work described how variation in early-expressed proteins Spalt and N peaks early in the related species *Junonia coenia* Hübner eyespot development [30], while later-expressed En displays overall lower temporal variation than Spalt and N. Such a pattern of variation is congruent with an hourglass model of eyespot development, although additional variation, in genes expressed after the hypothetical 'waist' stage, would need to be observed to support the model. Although our expression analyses focused on the developmental stage that is sensitive to environmental variation, the determination of eyespot size may be controlled by expression differences in genes other than those investigated here (i.e. those expressed after *Dll*) or at later time points in development (i.e. pupal development).

These results underscore the necessity for additional work on the functional genetics of eyespot development. While we show variation in the onset of gene expression between seasonal forms of *B. anynana*, the relationship between this variation and phenotypic plasticity of adult wings remains unclear. With the exception of Dll [19], [21], the function of the genes expressed in developing eyespots is not understood, and these roles may shift through development. Further understanding of the developmental mechanisms underlying plasticity in eyespot size will require additional functional data, along with an understanding of the upstream regulators and downstream targets of the N, En, and Dll proteins. Finally, the temporal dynamics of these and additional genes should be compared across species to determine whether gene expression timing during eyespot development is conserved among eyespot-bearing species.

## Supporting Information

Figure S1 **Observed temporal expression of Notch, Engrailed, and Distal-less, in the dry and wet season forms of** ***B. anynana*** **.** Graphs show frequency of expression type (central expression present or absent) for each developmental stage. Size of spot indicates relative number of samples at each developmental wing stage and lines are best-fit logistic curves for each gene and form.
(TIF)
