# Supplementary figures and images for: Temporal Gene Expression Variation Associated with Eyespot Size Plasticity in Bicyclus anynana
Source: PLoS One. 2013 Jun 10;8(6):e65830. doi: 10.1371/journal.pone.0065830 (PMC3677910; doi:10.1371/journal.pone.0065830)

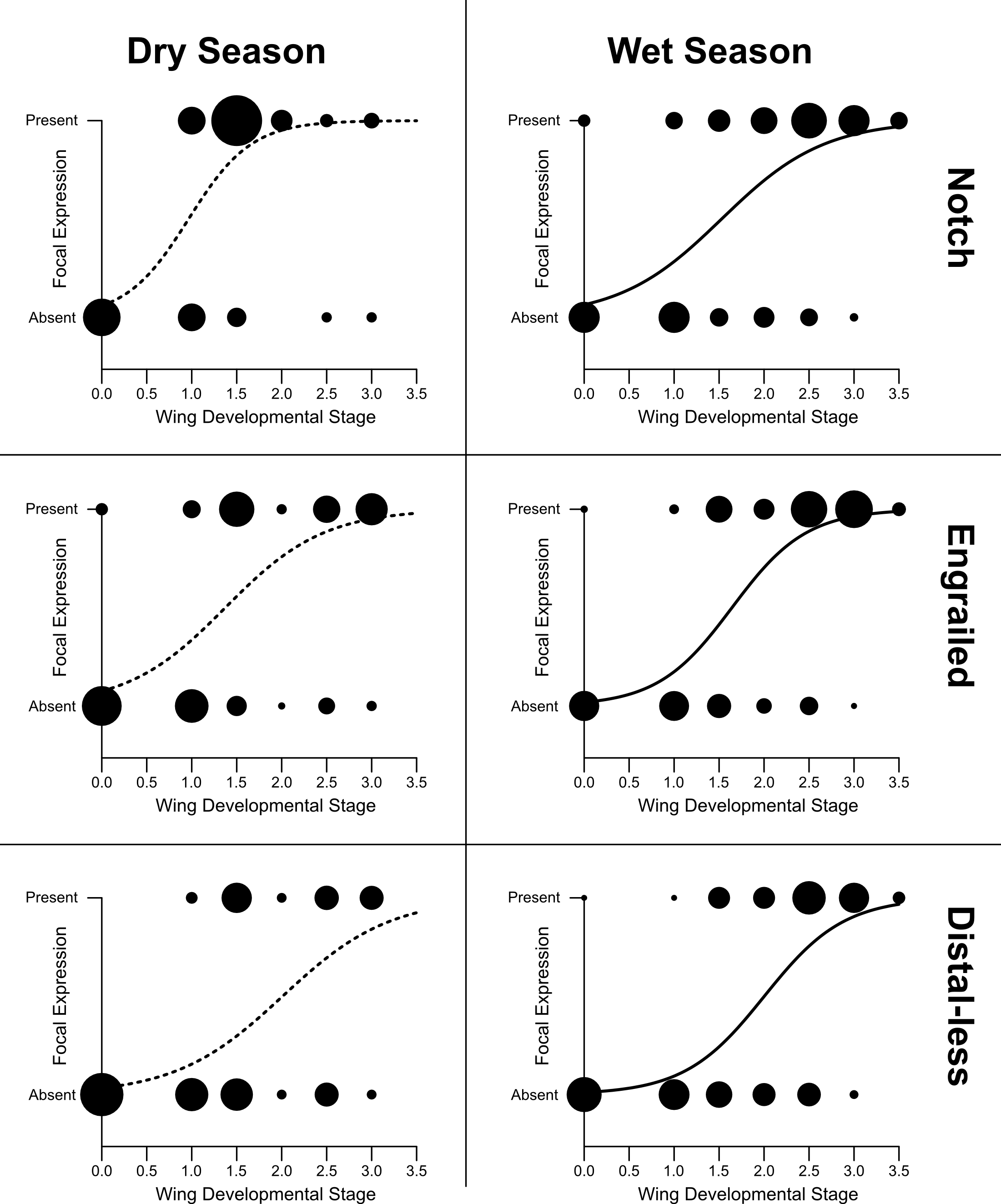

Supplement: Figure S1 — Observed temporal expression of Notch, Engrailed, and Distal-less, in the dry and wet season forms of B. anynana . Graphs show frequency of expression type (central expression present or absent) for each developmental stage. Size of spot indicates relative number of samples at each developmental wing stage and lines are best-fit logistic curves for each gene and form. (TIF) [file pone.0065830.s001.tif]
